# Comparison of Mouse Ly5^a^ and Ly5^b^ Leucocyte Common Antigen Alleles

**DOI:** 10.1155/1991/52686

**Published:** 1991

**Authors:** Suzanne L. Zebedee, Diana S. Barritt, William C. Raschke

**Affiliations:** La Jolla Biological Laboratories The Salk Institute P.O. Box 85350 San Diego California 92186 USA

## Abstract

The family of leucocyte common antigen (LCA) transmembrane glycoproteins is expressed in most hematopoietic cells. Molecular isoforms of the LCA molecule are generated by alternative splicing of a single gene encoded on the murine chromosome 1. Three LCA alleles with different antigenic reactivities have been identified in inbred mouse strains. To investigate the divergence between alleles, cDNA clones to the SJA (Ly5^a^) LCA gene have been isolated and sequenced. A comparison of this information to the Ly5^b^ allele sequence identifies 12 allele-specific nucleotide changes. These base substitutions correspond to five amino-acid changes within the extracellular domain of the LCA molecule. These amino-acid differences are clustered in a region that also contains the greatest divergence between mouse and rat LCA sequences. Thus, these two mouse LCA alleles exhibit a pattern of sequence conservation that mimics that found over a much broader scale of evolution. Analysis of antigenicity profiles for each of the allelic sequence changes reveals three molecular domains of altered antigenicity that could account for observed serological differences between the two alleles. Sequence information from the 5' end of the Ly5^a^ LCA gene, generated using polymerase chain-reaction techniques on genomic DNA, reveals eight additional nucleotide differences between the Ly5^a^ and Ly5^b^ alleles.


*Developmental Immunology*, 1991, Vol. 1, pp. 243–254
Reprints available directly from the publisher
Photocopying permitted by license only

© 1991 Harwood Academic Publishers GmbH
Printed in the United Kingdom

### Comparison of Mouse *Ly5*[a] and *Ly5*[b] Leucocyte Common Antigen Alleles

SUZANNE L. ZEBEDEE, DIANA S. BARRITT, and WILLIAM C. RASCHKE*

*La Jolla Biological Laboratories, The Salk Institute, P.O. Box 85350, San Diego, California 92186*

The family of leucocyte common antigen (LCA) transmembrane glycoproteins is expressed in most hematopoietic cells. Molecular isoforms of the LCA molecule are generated by alternative splicing of a single gene encoded on the murine chromosome 1. Three LCA alleles with different antigenic reactivities have been identified in inbred mouse strains. To investigate the divergence between alleles, cDNA clones to the SJA (*Ly5*[a]) LCA gene have been isolated and sequenced. A comparison of this information to the *Ly5*[b] allele sequence identifies 12 allele-specific nucleotide changes. These base substitutions correspond to five amino-acid changes within the extracellular domain of the LCA molecule. These amino-acid differences are clustered in a region that also contains the greatest divergence between mouse and rat LCA sequences. Thus, these two mouse LCA alleles exhibit a pattern of sequence conservation that mimics that found over a much broader scale of evolution. Analysis of antigenicity profiles for each of the allelic sequence changes reveals three molecular domains of altered antigenicity that could account for observed serological differences between the two alleles. Sequence information from the 5′ end of the *Ly5*[a] LCA gene, generated using polymerase chain-reaction techniques on genomic DNA, reveals eight additional nucleotide differences between the *Ly5*[a] and *Ly5*[b] alleles.

KEYWORDS: cDNA, domain, epitopes, evolution.

### INTRODUCTION

The leucocyte common antigen family of glycoproteins is abundantly expressed on the surface of most cells in the hematopoietic lineage (Scheid and Triglia, 1979; Sarmiento et al., 1982). These antigens (also known as CD45, *Ly5*, T200, and B220) have been identified in mouse, rat, chicken, and human systems (Komuro et al., 1975; Fabre and Williams, 1977; Judd et al., 1980; Omary et al., 1980; Houssaint et al., 1987). Data from the analysis of cDNA clones indicate that the murine LCA molecule has a hydrophobic leader sequence, an N-terminal extracellular domain consisting of 402-541 amino acids, a single transmembrane region, and a large cytoplasmic domain of 705 residues (Saga et al., 1986, with a correction, 1987; Thomas et al., 1987). The mapping of this family as a single gene on the mouse chromosome 1 has been completed and shown to comprise 34 exons (Saga et al., 1988); exons 1a and 1b are alternatively excluded 5′ untranslated sequences of LCA mRNAs, and exons 2–33 are protein–encoding. The function of the leucocyte common antigen has not been determined, although its involvement in leucocyte activities, such as natural killer cytolysis (Sparrow and McKenzie, 1983), cytotoxic T-cell cytolysis (Harp et al., 1984), as well as lymphocyte activation and differentiation (Yakura et al., 1986; Mittler et al., 1987; Pingel and Thomas, 1989) has been implicated. These roles may be mediated through the tyrosine phosphatase activity shown to be associated with the LCA molecule (Tonks et al., 1988).

In the mouse, the LCA molecule shows heterogeneity in molecular weight, glycosylation, and antigenicity patterns. Differences in LCA family members have been traced to the alternative splicing of exons 4, 5, and 6, which generates multiple molecular isoforms (Saga et al., 1987; Thomas et al., 1987). In B lymphoid cells, the major LCA protein product is 220,000 m.w. and contains all three of these alternative exons,

*Corresponding author.

whereas the major thymocyte protein is 180,000 m.w. and does not include exons 4, 5, or 6. These differences account for an insertion of 139 residues in the extracellular domain of the B-cell expressed LCA molecule. Proteins of intermediate size have been observed, which apparently represent splice combinations of exons 4, 5, and 6 (Chang et al., 1989). Glycosylation differences in the LCA protein family are also due to the inclusion of these variable exon sequences as the inserted amino acids contain many serine and threonine residues potentially linked to O-type carbohydrate structures (Childs et al., 1983; Johnson et al., 1989).

Murine LCA glycoproteins bear the *Ly5* alloantigenic determinant, which has been characterized in both inbred and wild strains of mice (Seldin et al., 1987). Inbred mice have been categorized into three LCA alleles, $Ly5^a$, $Ly5^b$, and $Ly5^c$, which can be distinguished antigenically by their reactivity with specific monoclonal and alloantibodies (*Ly5*.1, *Ly5*.2) and genetically by RFLPs of mouse genomic DNA (Seldin et al., 1987). Most established murine strains express the $Ly5^b$ allele, including BALB/c, C57BL, CBA/J, and NZB/BLNJ, yet SJL/J mice carry the $Ly5^a$ allele.

To examine the differences between the murine $Ly5^a$ and $Ly5^b$ alleles, we have isolated and analyzed cDNA clones from the $Ly5^a$ leucocyte common antigen allele. The differences between the two alleles provide a view through a narrow window of evolution at this locus, much narrower than that afforded by interspecies comparisons. The highly conserved as well as the more variable regions of the murine LCA molecules address the issues of functional importance and structural tolerance of these regions. The comparison also provides a better understanding of the antigenic differences that originally defined the alleles.

### MATERIALS AND METHODS

### Library Construction and Screening

Activated B and T cells were obtained by stimulating SJL/J.BALB/c-Ig$H^a$ (SJA) mouse spleen cells at $10^6$ cells/ml in RPMI 1640 medium, 10% fetal calf serum separately with LPS (25 $\mu$g/ml) or Con A (2 $\mu$g/ml). After 2 days in culture, cells were used to prepare poly A+ cytoplasmic RNA, as described previously (DeCino et al., 1988). A cDNA library was prepared using a mixture of activated SJA B- and T-cell mRNA according to standard procedures. First-strand synthesis was primed with a mixture of oligo $dT_{12-15}$ and random primers. Double-stranded fragments were prepared and cloned into the Lambda Zap II vector system (Stratagene Cloning Systems) using *Eco*RI linkers. Phage were grown, amplified, and screened according to procedures developed by the manufacturer. Positive phage were identified using synthetic oligonucleotides (Genetic Designs, Inc.) corresponding to the following nucleotide numbers of the $Ly5^b$ cDNA sequence (Saga et al., 1986, with a correction, 1987): oligonucleotide #18, 81–111; #19, 394–434; #7, 1426–1446; #10, 2784–2802; #17, 4563–4584.

### DNA Sequencing and Analysis

Purified phage were excised as pBluescript plasmids using procedures developed by the manufacturer (Stratagene) and confirmed to contain LCA sequences by restriction enzyme mapping and hybridization with the LCA-specific oligonucleotides described before. Plasmid DNA was purified on cesium-chloride density gradients (Ausubel et al., 1989) and sequenced by a modified dideoxy-chain-termination method (US Biochemicals) using Bluescript plasmid-specific primers and internal primers generated to the $Ly5^b$ LCA cDNA sequence (Saga et al., 1986, with a correction, 1987; Raschke, 1987).

Organization of the resulting DNA sequences and computer analysis was done on the Salk Institute VAX using University of Wisconsin Genetics Computer Group (UWGCG) programs (Devereux et al., 1984; Gribskov et al., 1986).

### Polymerase Chain Reaction

Cloned BALB/cke (Raschke, 1987) and SJA LCA cDNA samples were subjected to amplification by polymerase chain-reaction (PCR) procedures (Saiki et al., 1988) using a Perkin-Elmer Cetus DNA thermocycler and LCA-specific oligonucleotides. Messenger RNA prepared from Con A-stimulated SJA cells was copied using reverse transcriptase for 60 min at 37°C with specific antisense LCA primers, as described (Rappolee et al., 1989). Both plasmid (0.25 $\mu$g) and cDNA

samples (from 0.33 $\mu$g mRNA) were amplified for 35 PCR cycles in the presence of 1.5 U *Taq* polymerase (Perkin-Elmer Cetus), 250 ng each primer, 200 $\mu$M each dNTP in 1× buffer (50 mM KCl, 10 mM Tris, pH 8.3, 1.5 mM $MgCl_2$, and 0.1 mg/ml gelatin). One PCR cycle consisted of the following incubations: 30 sec at 94°C, 1 min at 50°C, and 5 min at 70°C. PCR products were extracted with phenol and chloroform, ethanol precipitated, treated with appropriate restriction enzymes, and separated by electrophoresis on 2% NuSieve, 1% agarose gels (FMC Corporation). For Southern blotting, DNA was transferred from the gel to ZetaProbe membranes (Bio-Rad) by capillary action in 0.4 M NaOH according to the manufacturer's instructions. Membranes were probed with $\gamma^{32}$P-ATP-labeled LCA oligonucleotides using ZetaProbe procedures and exposed to Kodak X-Omat film.

For cloning PCR-amplified genomic sequences, DNA was purified from SJL/J mouse blood samples as described (Ausubel et al., 1989) and amplified using LCA-specific oligonucleotides G44 and #46 (nucleotides 1–25 and 824–858, respectively, from Saga et al., 1988) as described before. Synthesis of the predicted 859-bp fragment was confirmed by agarose gel electrophoresis, and a portion (1/5) of the sample was reamplified by PCR. Following sample separation on an agarose gel, the 850-bp fragment was electroeluted, purified on a NACS column (Bethesda Research Laboratories), and kinased according to standard procedures (Ausubel et al., 1989). For cloning and sequencing of the genomic SJL fragment, the DNA was blunt-end ligated into the *Eco*RV site of the pBluescript vector polylinker (Stratagene Cloning Systems) and replicated in bacteria to prepare plasmid DNA. Sequencing of insert DNA was done as described before using Bluescript and LCA-specific primers.

### RESULTS

### Nucleotide and Predicted Amino-Acid Sequence of the *Ly5*[a] Leucocyte Common Antigen

To clone the leucocyte common antigen sequences corresponding to the murine *Ly5*[a] allele, a cDNA library to mRNA extracted from

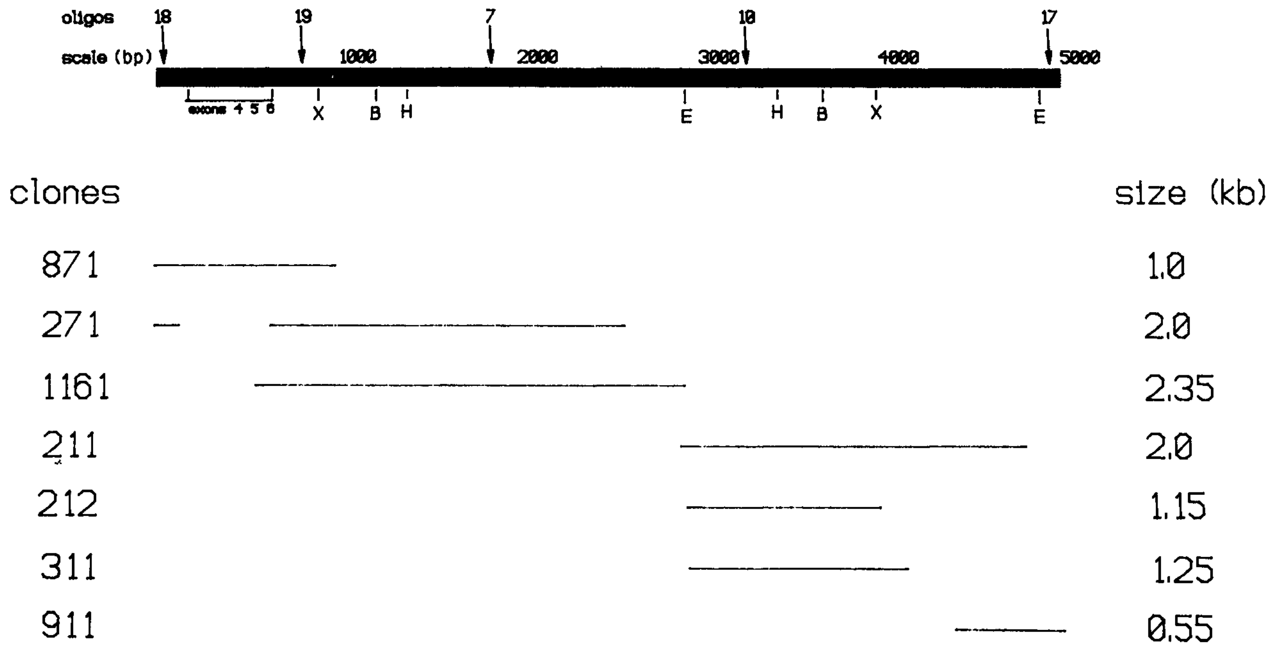

FIGURE 1. Clone organization of SJA leucocyte common antigen cDNAs. The cDNA clones selected from the SJA library are shown schematically as aligned with the B-cell *Ly5*[b] cDNA sequence (Saga et al., 1986, with a correction, 1987; Thomas et al., 1987). The oligonucleotides used for the clone selection (#18, 19, 7, 10, 17) and several restriction-enzyme cleavage sites (B, *Bam*HI; H, *Hin*dIII; E, *Eco*RI; X, *Xba*I) are indicated.

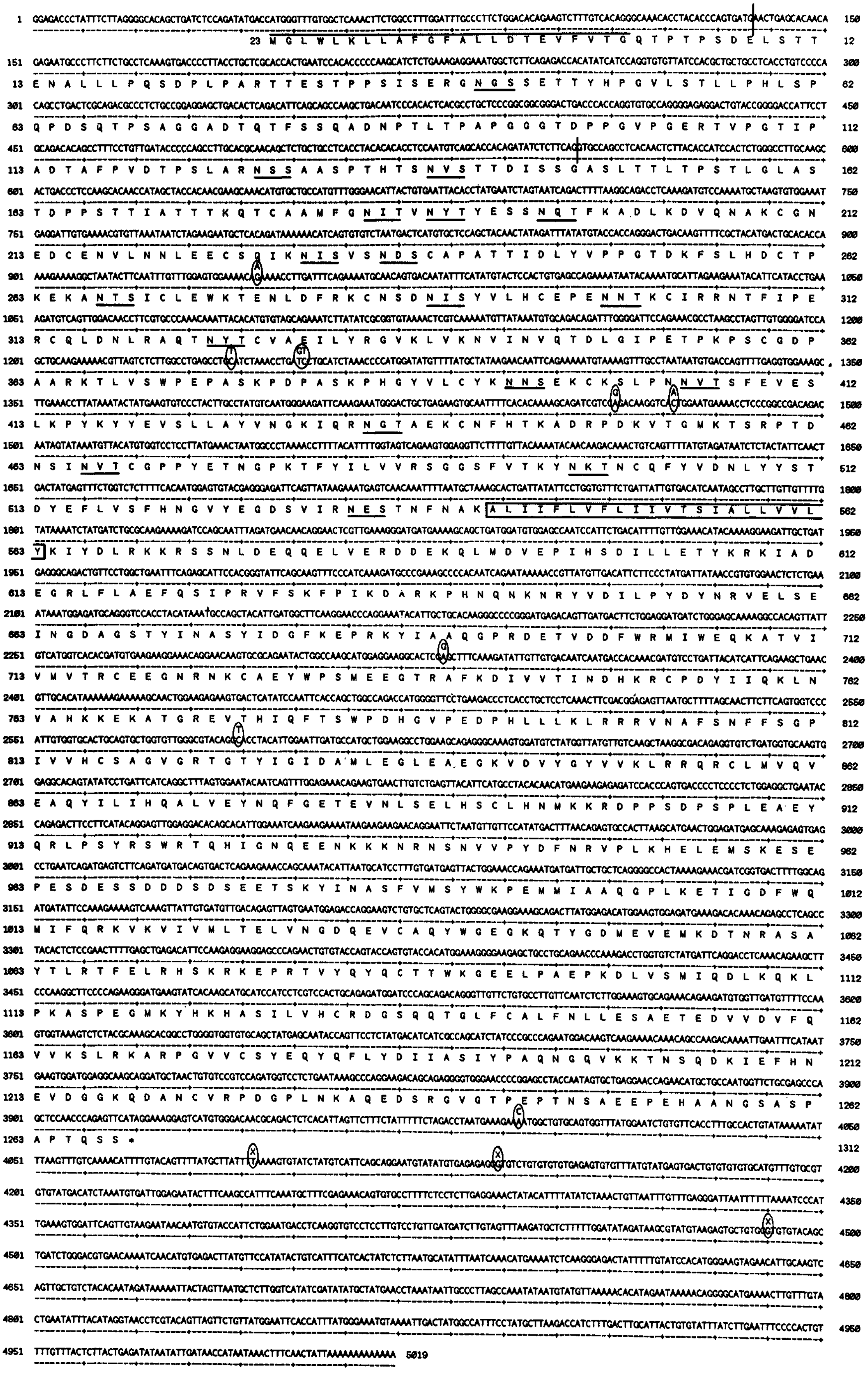

TABLE 1

Comparison of *Ly5*[a] and *Ly5*[b] Sequence Changes

| Nucleotide position[a] | Nucleotide | | Amino-acid position | Residue | | Protein domain[b] | Enzyme cleavage | |
|---|---|---|---|---|---|---|---|---|
| | *Ly5*[b] | *Ly5*[a] | | *Ly5*[b] | *Ly5*[a] | | *Ly5*[b] | *Ly5*[a] |
| 943 | A | G | 277 | K | E | Extracellular | — | — |
| 1238 | T | C | 375 | V | A | Extracellular | — | SfaNI |
| 1251 | G | T | 379 | E | D | Extracellular | — | Sau3A |
| 1252 | T | C | 380 | S | P | Extracellular | HinfI | — |
| 1461 | G | A | 449 | — | | Extracellular | HpaII | — |
| 1472 | A | C | 453 | N | T | Extracellular | — | — |
| 2328 | G | A | 738 | — | | Cytoplasmic | — | XhoI |
| 2589 | T | C | 825 | — | | Cytoplasmic | KpnI | — |
| 3993 | C | A | — | — | | 3' untranslated | — | — |
| 4081 | — | T | — | — | | 3' untranslated | — | — |
| 4139 | — | G | — | — | | 3' untranslated | — | — |
| 4490 | — | G | — | — | | 3' untranslated | — | — |

[a]Nucleotide position corresponding with the first nucleotide of exon 2 as number 1.
[b]For nucleotide differences in the 5' untranslated region, see Fig. 4. No nucleotide changes for the *Ly5*[a] and *Ly5*[b] alleles were observed in the predicted signal or transmembrane regions.

mitogen-stimulated SJA mouse spleen cells was prepared. The cDNA clones were inserted into lambda phage system vectors (see Materials and Methods) and screened with oligonucleotide probes derived from the published *Ly5*[b] sequence (Saga et al., 1986, with a correction, 1987; Raschke, 1987). A profile of seven isolated LCA-specific clones and the oligonucleotides used for their screening is diagrammed in Figure 1. These clones were subjected to nucleic-acid sequence analysis and found to contain overlapping information from the entire exon arrangement of the murine LCA gene (Saga et al., 1988). Clones 871 and 271 contain information from the 5′ untranslated end of the LCA gene: 871 is initiated from exon 1a, whereas the 5′ end of clone 271 is from the alternative exon 1b. These clones also include different sequence information from the alternatively spliced B-cell isoform exons (Thomas et al., 1987). Sequence from exons 4, 5, and 6 is contained in clone 871; clone 271 is derived from an alternatively processed mRNA, which is missing these three exons.

Figure 2 shows the organized nucleotide sequence of the overlapping *Ly5*[a] cDNAs and the predicted amino-acid sequence generated from the single large open reading frame. The 5′ signal sequence (23 amino acids), single membrane-spanning domain (22 amino acids), and orientation of the protein are predicted from hydropathy plots and homology with previously published LCA sequences (Thomas et al., 1985; Saga et al., 1986, with a correction, 1987). These comparisons predict a 541 residue extracellular domain for the B-cell isoform (clone 871, as shown in Figure 2), which contains information for an additional 139 amino acids than the T-cell form (clone 271, indicated by vertical marks in Figure 2). A cytoplasmic domain of 705 amino acids is predicted for the murine *Ly5*[a] molecule from these sequences.

### Comparison of *Ly5*[a] and *Ly5*[b] Sequences

Nucleotide differences between the SJA *Ly5*[a] cDNA sequence and the published mouse *Ly5*[b] sequence (Saga et al., 1986, with a correction, 1987; Raschke, 1987; Thomas et al., 1987) are also shown in Figure 2. This sequence comparison reveals nine single nucleotide substitutions and three nucleotide additions in the cloned *Ly5*[a] sequence. Table 1 summarizes these nucleotide

FIGURE 2. Nucleotide sequence of the SJA leucocyte common antigen. The nucleotide sequence of the isolated SJA cDNA clones (*Ly5*[a]) as determined by dideoxy-chain-termination sequencing is shown, as is the translation into single-letter amino-acid abbrevations. Sequence differences between the SJA *Ly5*[a] allele and the BALB/c *Ly5*[b] allele (Saga et al., 1986, with a correction, 1987; Raschke, 1987; Thomas et al., 1987) are indicated with the *Ly5*[b] difference shown directly above the *Ly5*[a] nucleotide (X indicates no nucleotide) and are encircled. The predicted LCA-signal sequence is indicated by a line over the amino-acid translation, the membrane-spanning region is denoted by a box, and potential asparagine-linked glycosylation sites are underlined. The inserted 5' B-cell sequences included in clone 871, but not in 271, are within the indicated vertical lines. For presentation ease, the nucleic-acid sequence commences with the first nucleotide of exon 2 (Saga et al., 1988), although clones 871 and 271 contain sequence information from exons 1a and 1b (see text and Figure 4).

changes and the resulting amino-acid differences. Five of the nucleotide substitutions result in an amino-acid change of the LCA protein between the *Ly5*[a] and *Ly5*[b] alleles: residue 277, lysine to glutamate; residue 375, valine to alanine, residue 379, glutamate to aspartate; residue 380, serine to proline; and residue 453, asparagine to threonine. As shown in Table 1, other nucleotide changes between the two sequences either do not result in a residue alteration, or they occur in the 3′ untranslated region of the cDNA.

All of the amino-acid differences between the two murine LCA alleles occur in the extracellular domain of the protein, and no changes are observed in the transmembrane or cytoplasmic regions. Differences in the extracellular protein composition are amino-acid substitutions only, and do not change any of the potential N-linked glycosylation sites, nor do they affect the B-cell insert exons 4, 5, and 6. One substitution, at residue 277, alters the charge of the LCA protein [lysine (+) to glutamate (—)] between *Ly5*[a] and *Ly5*[b]. Other substitutions are conservative (valine to alanine; glutamate to aspartate) or involve changes in hydrophobicity and protein structure (serine to proline; asparagine to threonine), as indicated in Table 1.

### Confirmation of Nucleotide Sequence using PCR

Several of the nucleotide changes between the *Ly5*[a] and *Ly5*[b] alleles result in the loss or acquisition of restriction enzyme sites, as indicated in Table 1. To confirm these nucleotide differences, both plasmid DNA and mRNA from both LCA alleles were subjected to polymerase chain-reaction (PCR) amplification using LCA-specific oligonucleotides surrounding each change to be tested. The amplified products from both *Ly5*[a] and *Ly5*[b] sequences were then digested with the restriction enzymes from Table 1 and examined for the loss or addition of individual sites. Figure 3 shows the resulting PCR cleavage patterns for two *Ly5*-specific sites: *Hin*fI at nucleotide 1252 of the *Ly5*[b] sequence and *Kpn*I at nucleotide 2589 of *Ly5*[b]. In both cases, DNA from amplified *Ly5*[b] samples is digested in a predictable manner,

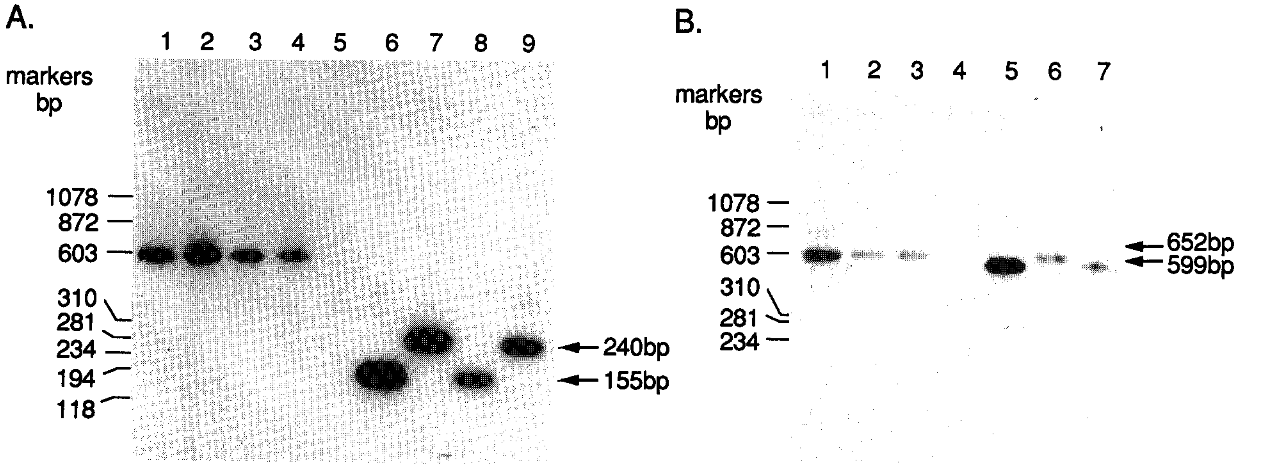

FIGURE 3. PCR analysis of nucleotide-sequence differences by enzyme cleavage. Nucleotide-sequence differences between *Ly5*[a] and *Ly5*[b] were detected from PCR-amplified cDNA and mRNA samples using *Ly5*[b]-specific cleavage sites for *Hin*fI (A) at nucleotide position 1251 and *Kpn*I (B) at position 2589. Digestion patterns were detected from separation on agarose gels, transfer to ZetaProbe membranes, and detection with oligonucleotide probes. (A) cDNA (lanes 1, 2, 6, 7) or mRNA (lanes 3, 4, 8, 9) corresponding to the *Ly5*[b] (lanes 1, 3, 6, 8) or *Ly5*[a] (lanes 2, 4, 7, 9) sequence were PCR-amplified using LCA-specific olgionucleotides at positions #1114–1131 and #1657–1669 (Fig. 2). Amplified samples were digested with *Hin*fI (lanes 6–9) or not treated (lanes 1–4), and probed with an LCA oligonucleotide (#1339–1358), which is predicted to detect a *Hin*fI cleavage product of 155 bp from the *Ly5*[b] sequence or 240 bp from the *Ly5*[a] sequence. Undigested DNA is expected to yield a 555-nucleotide fragment. Lane 5 shows a sample containing water in place of DNA or RNA subjected to PCR amplification. (B) Samples from *Ly5*[b] cDNA (lanes 1, 5; Raschke, 1987) or mRNA (lanes 3, 7) and *Ly5*[a] mRNA (lanes 2, 6) were PCR-amplified with oligonucleotides corresponding to nucleotides #2531–2567 and #3167–3186 of LCA sequences (Fig. 2). The resulting *Kpn*I digestion of these samples (lanes 5–7) and detection with an internal oligonucleotide (#2955–2979) are shown. Only the *Ly5*[b] sequence is predicted to produce a PCR product that can be cleaved from 652 bp to 599 bp with *Kpn*I. Lane 4 shows a sample containing water in place of DNA or RNA subjected to PCR amplification.

TABLE 2

Structural and Antigenic Comparison of Altered *Ly5*[a] and *Ly5*[b] Domains

| Position[a] | Amino-acid residue | | Chou–Fasman prediction[b] | | Antigenicity index[c] | |
|---|---|---|---|---|---|---|
| | *Ly5*[a] | *Ly5*[b] | *Ly5*[a] | *Ly5*[b] | *Ly5*[a] | *Ly5*[b] |
| 275 | K | K | h | h | 0.900 | 0.900 |
| 276 | T | T | h | h | 0.900 | 1.300 |
| 277* | E | K | h | h | 0.900 | 1.300 |
| 278 | N | N | h | h | 0.900 | 1.300 |
| 279 | L | L | h | h | 0.750 | 1.150 |
| 280 | D | D | h | h | 1.150 | 1.150 |
| 371 | W | W | B | B | 0.300 | 0.300 |
| 372 | P | P | t | t | 0.800 | 0.650 |
| 373 | E | E | t | t | 1.100 | 0.950 |
| 374 | P | P | — | — | 1.300 | 0.900 |
| 375* | A | V | t | — | 1.500 | 0.900 |
| 376 | S | S | t | — | 1.100 | 0.900 |
| 377 | K | K | — | — | 0.900 | 0.900 |
| 378 | P | P | T | t | 1.300 | 1.100 |
| 379* | D | E | T | t | 1.300 | 1.100 |
| 380* | P | S | T | — | 1.300 | 0.900 |
| 381 | A | A | T | t | 1.300 | 1.100 |
| 382 | S | S | t | t | 1.100 | 1.100 |
| 450 | D | D | T | T | 1.300 | 1.300 |
| 451 | K | K | h | — | 0.900 | 0.900 |
| 452 | V | V | h | t | 0.750 | 0.950 |
| 453* | T | N | h | t | 0.750 | 1.500 |
| 454 | G | G | h | — | 0.750 | 1.150 |
| 455 | M | M | h | — | 0.600 | 1.000 |
| 456 | K | K | h | — | 0.900 | 0.900 |
| 457 | T | T | — | — | 0.900 | 0.900 |

[a]Altered amino-acid positions between *Ly5*[a] and *Ly5*[b] are indicated by an asterisk (*).
[b]Chou and Fasman, 1978 h=helix, B=sheet, and t, T=turn regions, with T>t for strength of prediction.
[c]Jameson and Wolf, 1988.

whereas *Ly5*[a] sequences are not cleaved at these specific sites (Figure 3). Similarly, an analysis of amplified DNA from LCA clones and mRNA at the other enzyme sites (e.g., *SfaNI*, *Sau*3A, *Hpa*II, and *Xho*I) also resulted in the appropriate fragment digestion patterns (data not shown). Thus, the nucleotide sequence differences between these two LCA alleles at 6 of the 12 altered sites have been confirmed.

### Antigenic Analysis of LCA Allele Differences

To examine the correlation between the observed molecular differences in amino-acid sequence and the antigenic variation of the *Ly5*[a] and *Ly5*[b] proteins, a computer analysis of antigenicity was performed. The amino-acid sequence corresponding to the extracellular and membrane-spanning domains of each LCA molecule was examined for alterations in the antigenic index (Jameson and Wolf, 1988), which includes for each amino-acid residue measurements of hydrophobicity, surface probability, flexibility, and secondary structure. The resulting data indicate significant differences in antigenicity (values >1.2) between the *Ly5*[a] and *Ly5*[b] leucocyte common antigen alleles across the regions of amino-acid change (Table 2). A comparison of the corresponding figures for Chou–Fasman predictions (Chou and Fasman, 1978) and antigenic index (Jameson and Wolf, 1988) reveals three domains of altered antigenicity and structure around the following amino acids: residue 277, increased antigenicity of the *Ly5*[b] protein; residues 375–380, increased antigenicity and structural alteration of *Ly5*[a]; and residue 453, increased antigenicity and predicted structural change of the *Ly5*[b] molecule.

### Nucleotide Sequence of *Ly5*[a] Genomic DNA

The isolated SJA cDNA clones contain a portion of the complete sequence information available for the *Ly5*[b] exons 1a and 1b sequence (Saga et al., 1988). To obtain the remaining nucleotide

     501  TGAATTAAGGAAGTAAGAAGCCATTGCACTGACTTTGAACGACCTTTTTTTTTTTTTTTTTAACTTCCTGCAAAGAGGACCCTTTACAGTATTTTTGGAG  600

                                                   X                         A

     601  AAGTTAGTAAAACCGAATCTGACATCACCATTTAGCAGTGCATGTAGCTAGCAAGTGGTTTGTTCTTAGGGTAAGAGAGTAGGAAACTTGCTCCCCATCT  700

                              Exon 1b →

                                           G

     701  GATAAGACAGAGTGCAAAGTATGCGTTCTTTTCTTTTAGTTTTGACTTAGCTTTACAGAGACAAACTTCAAGAGAGATAACCATTATTTTGCCTTTCAGG  800

                 clone 271  End 1b

     801  GAGACCCTATTTCTTAGGGGCACAGCTGATCTCCAGATATGACCATGGGTTTGTGGCTC  859
          Exon 2 →                          Oligo #46
```

FIGURE 4. Nucleotide sequence of SJL/J genomic DNA including exons 1a and 1b. The nucleotide sequence of the fragment generated by PCR using SJL genomic DNA and the G44 and #46 oligonucleotides is shown. Differences with the *Ly5*[b] sequence (Saga et al., 1988) are indicated above the *Ly5*[a] sequence (X indicates no nucleotide). The LCA-specific oligonucleotides used for fragment generation (G44, #46) are boxed, and the information obtained from cDNA library isolated clones (871, 271) is underlined. The start and end of exons 1a and 1b sequences (Saga et al., 1988) are shown. The start of exon 2 is also indicated and corresponds to the beginning of the cDNA sequence shown in Fig. 2. Oligonucleotide 46 lies within exon 2. The string of thymidines that showed some variation in length between individual clones of PCR generated fragments is indicated by a heavy underline.

sequence from the 5′ end of the LCA mRNA molecules and to examine the potential transcription regulatory sequences, a genomic fragment that includes exons 1a and 1b sequences was generated from *Ly5*[a] DNA using PCR techniques. Genomic DNA was isolated from SJL/J (*Ly5*[a]) blood samples and amplified using LCA oligonucleotides G44 and #46 prepared to the *Ly5*[b] published sequence at the 5′ gene end (Figure 4; Saga et al., 1988). The nucleotide sequence of the resulting 859-bp PCR product is shown in Figure 4 as compared to the *Ly5*[b] genomic sequence. Eight nucleotide differences in this region of the LCA gene have been identified between these two murine alleles. Two changes affect the sequence of exon 1b: an insertion of a T at position 41, and a deletion of an A at position 64 of the exon 1b *Ly5*[a] sequence. As indicated in Figure 4, cDNA clone 271 does not extend far enough to detect these changes in exon 1b. Six other nucleotide differences are present between the BALB/c and SJL LCA genomic fragment, yet none of these changes is expected to affect LCA gene regulatory TATA-like sequences (Saga et al., 1988), gene splicing, or coding arrangements. This PCR-generated *Ly5*[a] genomic sequence was confirmed from separate fragments isolated from independent amplification reactions, with the exception of one stretch of 17 thymidines in the intron between exons 1a and 1b. Several of the individually cloned PCR fragments contained 15 or 16 thymidines, indicating a tendency of the amplification reaction to unfaithfully reproduce stretches of identical nucleotides. Although the possibility exists that the *Ly5*[a] gene has fewer than 17 thymidines in this location, the sequence shown in Figure 4 with 17 thymidines was found in the majority of the fragments analyzed.

### DISCUSSION

The nucleotide and amino-acid sequence of the leucocyte common antigen from a second murine allele (*Ly5*$^a$) is reported. A comparison of this cDNA sequence with that of the published *Ly5*$^b$ allele (Saga et al., 1986, with a correction, 1987; Thomas et al., 1987) reveals 12 nucleotide changes. Several of these nucleotide changes cause alterations in the restriction enzyme digest patterns of LCA cDNA (see Table 1), and these enzymes were used to detect and confirm the specific changes among the two alleles by PCR using LCA-specific oligonucleotides. Five amino-acid substitutions between *Ly5*$^a$ and *Ly5*$^b$ result from the allele nucleotide differences, and these changes all occur in the extracellular domain of the membrane-spanning LCA molecule. Interestingly, this variation in the extracellular domain of the two murine alleles parallels the homology found among interspecies LCA molecules. The LCA extracellular region is only about 50% similar among human, rat, and mouse LCA molecules (Tung et al., 1988), whereas the transmembrane and cytoplasmic regions show a higher degree of homology among these species (80–90%). Therefore, the same selection pressures are already evident over the short evolutionary time scale.

The accumulation of differences in the various regions of the *Ly5* gene can be considered indicative of the tolerance of changes in these areas with respect to expression of the gene or function of the gene product. Other than the regions encoding the leader sequence and the transmembrane regions that are comparatively conserved, the cytoplasmic domain has the lowest frequency of differences, 0.09% (2 differences/2115 base pairs). The extracellular domain and 3′ untranslated region each have a frequency of 0.37% (6/1623 and 4/1085, respectively). The greatest density of differences between the *Ly5*$^a$ and *Ly5*$^b$ regions sequenced is at the 5′ end, where the transcribed but untranslated sequences (exons 1a and 1b and part of exon 2) have a frequency of 0.75% (2/268) and the genomic, untranscribed sequences have a frequency of 1.04% (6/576).

The significance of the greater conservation of noncoding sequences at the 3′ end of the gene compared to the 5′ end is not clear, although a role for the 3′ untranslated sequence in the stability of the mRNA is possible. None of the nucleotide differences in the 5′ genomic sequences of the two alleles (Figure 4) is predicted to affect transcript initiation or the start of translation for this gene. It should be noted that the *Ly5*$^a$ genomic sequence was generated from independent clones using polymerase chain reaction techniques reported to have a nucleotide incorporation-error frequency of 0.25% (Saiki et al., 1988). In this case, the only examples of enzyme infidelity occurred in a stretch of 17 thymidines in which some of the cloned PCR fragments contained fewer thymidines.

Conservation of the 705 amino-acid cytoplasmic domain between the two mouse alleles and among other species implies a functional importance for this region of the LCA molecule. Features of the leucocyte common antigen, including the size and conservation of the cytoplasmic domain, cytoplasmic phosphorylated residues (Thomas et al., 1985; Shackelford and Trowbridge, 1986), cytoplasmic protein tyrosine phosphatase activity (Tonks et al., 1988; Ostergaard et al., 1989), and association with the cytoskeletal fodrin molecule (Bourguignon et al., 1985), have led to the speculation that this family of molecules is involved in signal transduction. It has been demonstrated that the LCA molecule regulates T-cell growth (Kiener and Mittler, 1989; Ostergaard et al., 1989; Pingel and Thomas, 1989) through changes in molecular phosphorylation pathways. This hematopoietic cell-growth regulation may be triggered through the interaction of LCA with different T- and B-cell receptor molecules (Ledbetter et al., 1988) or soluble ligands as mediated by the different cell-type-specific LCA isoforms. In this matter, the isoforms could account for the variety of activities observed for the LCA molecule, such as lymphocyte differentiation and proliferation, T-cell cytolysis, and natural killer activity.

The nucleotide and amino-acid differences between the two alleles in the extracellular domain are not evenly distributed, but occur within a 530-bp, 177 amino-acid segment in the second half of the domain. The first half and last sixth of the extracellular domain are as conserved as the cytoplasmic domain at the protein level for these two murine *Ly5* alleles. Interestingly, the region of differences in the extracellular domain is also the region with the most variation between the rat (Barklay et al., 1987; Rappolee et al., 1989) and mouse (Saga et al., 1986, with a correction, 1987; Raschke, 1987) LCA extracellular sequences, having an amino-acid homology in this region of 51% compared with 67% for the remainder of the domain.

The comparison of the rat LCA sequence with the mouse alleles reveals some interesting relationships. Of the 12 nucleotide differences in the cDNAs of the *Ly5* alleles, the *Ly5*$^a$ and rat sequences are identical at four of the positions (nucleotides 1238, 1461, 2589, and 4092; see Figure 2 and Table 1). In another three of the positions (nucleotides 943, 1472, and 2328), the *Ly5*$^b$ nucleotide is identical to that in the rat sequence. [Of the remaining five positions of allelic sequence differences, two (nucleotides 1251 and 1252) are in an 18-nucleotide segment not present in the rat and one (nucleotide 4490) is in a region for which the rat sequence is not published.] The amino acids encoded by these differences are also identical between rat and the respective murine allele, in spite of the other numerous species differences in this region at the nucleotide and amino-acid levels. Thus, at *each Ly5* allelic difference in the *coding* region, the corresponding rat nucleotide is identical to either the *Ly5*$^a$ or *Ly5*$^b$ nucleotide, with the exception of the two located in a segment that is absent in the rat sequence. This relationship is unlikely to be fortuitous, yet no clear explanation based on evolutionary progression or selection is evident.

Two clones were isolated from the SJA library with distinct 5′ sequence information from exons 1a and 1b, and the B-cell insert exons 4, 5, and 6. The cDNA library was prepared to mRNA from a mixture of mitogen-stimulated B- and T-cell populations in order to obtain B- and T-cell sequence information for the *Ly5*$^a$ allele. Various alternative splice combinations of exons 4, 5, and 6 have been observed among LCA family members (reviewed in Thomas and Lefrancois, 1988), including the identification of mRNAs corresponding to six of the eight possible alternatively processed transcripts. Our screening of the *Ly5*$^a$ library using 5′ oligonucleotides identified only two of these combinations: one with exons 4+5+6, and the other without any of these exons. The association of exon 4, 5, and 6 splice patterns with transcript initiation from exon 1a or 1b for the LCA molecule does not appear to be cell-type-specific (Saga et al., 1987, 1988). In fact, the isolated clones described contain exon 1a with the B-cell insert of exons 4, 5, and 6 (clone 871) and exon 1b without exons 4, 5, and 6 (clone 271), which is opposite of the transcript organization that appears to be most abundant for this gene family (Saga et al., 1988).

Determination of the amino-acid sequence for the leucocyte common antigen *Ly5*$^a$ allele allows for an examination of the alloantigenic distinctions from the murine *Ly5*$^b$ allele on a molecular level. Changes in the nucleotide sequence that result in different restriction enzyme patterns (Table 1) for these two murine alleles can now be used in combination with PCR techniques as an allele-specific assay system. Previous distinctions between these alleles have relied upon proteolysis experiments, RFLP patterns, and antigenic reactivities. Cleveland peptide maps generated with V8 protease (Tung et al., 1981) can now be explained by the amino-acid changes at glutamate residues (see Table 1) between these two alleles. Also, conventional *Ly5* alloantisera (prepared from the immunization of intraallelic F1 mice with cells from the other allele; Scheid and Triglia, 1979) and allele-specific monoclonal antibodies (Shen, 1981) have been shown to distinguish mice carrying the *Ly5*$^a$ (*Ly5*.1 antisera) or *Ly5*$^b$ (*Ly5*.2 antisera) alleles (Seldin et al., 1987). Analysis of the antigenicity of the *Ly5*$^a$ versus *Ly5*$^b$ alleles based on the amino-acid composition of the extracellular region highlights three major antigenic differences at residues 276–279, 372–381, and 451–456 (Table 2). Reactivities of the LCA alleles with alloantisera are likely to be due to recognition differences of the amino acids and molecular structure of these domains. The antigenic index for the *Ly5*$^a$ allele becomes higher in value at only one of these regions (residues 372–381, see Table 2), thus suggesting that this is the major epitope for the *Ly5*.1 antibodies. The other two allele-distinct LCA domains show increased antigenicity of the *Ly5*$^b$ allele; therefore, the monoclonal and alloantibody recognition of this allele product is predicted to be through one or both of these epitopes. Interestingly, as both *Ly5*$^a$ and *Ly5*$^b$ mice express an apparently functional LCA protein, the identification of distinct allele-specific extracellular residues suggests that these regions are not likely to be involved in a common cell function for the LCA molecule.

Allele-specific differences in the murine leucocyte common antigen have been identified from cDNA cloning and sequence analysis. The observed nucleotide changes between SJA (*Ly5*[a]) and BALB/c (*Ly5*[b]) mice are predicted to be allele-specific, although additional differences between SJA and other *Ly5*[a] mice, as well as BALB/c and other *Ly5*[b] mice may occur. Additional intraallelic changes are not expected to play a role in the antigenicity differences between the *Ly5*[a] and *Ly5*[b] alleles. Changes in the nucleic-acid sequence among these two alleles can be used to categorize mouse mRNA or DNA into *Ly5*[a] or *Ly5*[b] subtypes using PCR and sequence-specific restriction-enzyme cleavage sites. The identified nucleotide-sequence changes translate into extracellular protein domains distinguished by antibody reactivity, for which a molecular basis is now proposed.

### ACKNOWLEDGMENTS

The authors would like to thank Patricia Koutz and Barbara Fortanely for technical assistance, and Irv Edelman at Genetics Computer Group for his help with sequence antigenicity profiles. This work was supported by National Institutes of Health grant GM32017.

*(Received October 10, 1990)*

*(Accepted January 16, 1991)*

### REFERENCES

Ausubel F.M., Brent R., Kingston R.E., Moore D.D., Seidman J.G., Smith J.A., and Struhl K. (1989) *In*: Current Protocols in Molecular Biology, (New York: John Wiley & Sons).

Barklay A.N., Jackson D.I., Willis A.C., and Williams A.F. (1987). Lymphocyte specific heterogeneity in the rat leucocyte common antigen (T200) is due to differences in polypeptide sequences near the $NH_2$-terminus. EMBO J. **6:** 1259.

Bourguignon L.Y.W., Suchard S.J., Nagpal M.L., and Glenney J.R. (1985). A T-lymphoma transmembrane glycoprotein (gp180) is linked to the cytoskeletal protein, fodrin. J. Cell. Biol. **101:** 4877–4897.

Chang H.-L., Zarovkian M.H., and Esselman W.J. (1989). T200 alternate exon use in murine lymphoid cells determined by reverse transcription-polymerase chain reaction. J. Immunol. **143:** 315–321.

Childs R.A., Dalchau R., Scudder P., Hounsell E.F., Fabre J.W., and Feizi T. (1983). Evidence for the occurrence of O-glycosidically linked oligosaccharides of poly-N-acetyllactosamine type on the human leucocyte common antigen. Biochem. Biophys. Res. Comm. **110:** 424–431.

Chou D.Y., and Fasman G.D. (1978). Empirical predictions of protein conformation. Ann. Rev. Biochem. **47:** 251–276.

DeCino P., Lernhardt W., Herbst H., and Raschke W.C. (1988). Expression of oncogenes in normal and transformed murine B lymphocytes. Oncogene Res. **3:** 33–37.

Devereux J., Haeberli P., and Smithies O. (1984). A comprehensive set of sequence analysis programs for the VAX. Nucleic Acids Res. **12:** 387–395.

Fabre J.W., and Williams A.F. (1977). Quantitative serological analysis of a rabbit anti-rat lymphocyte serum and preliminary biochemical characterization of the major antigen recognized. Transplantation **23:** 349–359.

Gribskov M., Burgess R.R., and Devereux J. (1986). Pepplot, a protein secondary structure analysis program for the UWGCG sequence analysis software package. Nucleic Acids Res. **14:** 327–334.

Harp J.A., Davis B.S., and Ewald S.J. (1984). Inhibition of T cell responses to alloantigens and polyclonal mitogens by Ly-5 antisera. J. Immunol. **133:** 10–15.

Houssaint E., Tobin S., Cihak J., and Losch U. (1987). A chicken leucocyte common antigen: Biochemical characterization and ontogenetic study. Eur. J. Immunol. **17:** 287–290.

Jameson B.A., and Wolf H. (1988). The antigenic index: A novel algorithm for predicting antigenic determinants. CABIOS **4:** 181–186.

Johnson N.A., Meyer C.M., Pingel J.T., and Thomas M.L. (1989). Sequence conservation in potential regulatory regions of the mouse and human leukocyte common antigen gene. J. Biol. Chem. **264:** 6220–6229.

Judd W., Poodry C.A., Broder S., Friedman S.M., Chess L., and Strominger J.L. (1980). High molecular weight antigens present on human T cells. Proc. Natl. Acad. Sci. USA **77:** 6805–6809.

Kiener P.A., and Mittler R.S. (1989). CD45-protein tyrosine phosphatase cross-linking inhibits T cell receptor CD3-mediated activation in human T cells. J. Immunol. **143:** 23–28.

Komuro K., Itakura K., Boyse E.A., and John M. (1975). Ly-5: A new T-lymphocyte antigen system. Immunogenetics **1:** 452–456.

Ledbetter J.A., Tonks N.K., Fischer E.H., and Clark E.A. (1988). CD45 regulates signal transduction and lymphocyte activation by specific association with receptor molecules on T or B cells. Proc. Natl. Acad. Sci. USA **85:** 8628–8632.

Mittler R.S., Greenfield R.S., Schacter B.Z., Richard N.F., and Hoffman M.K. (1987). Antibodies to the common leukocyte antigen (T200) inhibit an early phase in the activation of resting human B cells. J. Immunol. **138:** 3159–3166.

Omary M.B., Trowbridge I.S., and Battifora H.A. (1980). Human homologue of murine T200 glycoprotein. J. Exp. Med. **152:** 842–852.

Ostergaard H.L., Shackelford D.A., Hurley T.R., Johnson P., Hyman R., Sefton B.M., and Trowbridge I.S. (1989). Expression of CD45 alters phosphorylation of the *Lck*-encoded tyrosine protein kinase in murine lymphoma T-cell lines. Proc. Natl. Acad. Sci. USA **86:** 8959–8963.

Pingel J.T., and Thomas M.L. (1989). Evidence that the leucocyte-common antigen is required for antigen-induced T lymphocyte proliferation. Cell **58:** 1055–1065.

Rappolee D.A., Wang A., Mark D., and Werb Z. (1989). Novel method for studying mRNA phenotypes in single or small numbers of cells. J. Cell. Biochem. **39:** 1–8.

Raschke W.C. (1987). Cloned murine T200 (*Ly5*) cDNA reveals multiple transcripts within B and T-lymphocyte lineages. Proc. Natl. Acad. Sci. USA **84:** 161–165.

Saga Y., Tung J.-S., Shen F.-W., and Boyse E.A. (1986). Sequences of *Ly5* cDNA: Isoform-related diversity of *Ly5* mRNA. Proc. Natl. Acad. Sci. USA **83:** 6940–6944. With a correction: (1987). Proc. Natl. Acad. Sci. USA **84:** 1991.

Saga Y., Tung J.-S., Shen F.-W., and Boyse E.A. (1987). Alternative use of 5′ exons in the specification of *Ly5* isoforms distinguishing hematopoietic cell lineages. Proc. Natl. Acad. Sci. USA **84:** 5364–5368.

Saga Y., Tung J.-S., Shen F.-W., Pancoast T.C., and Boyse E.A. (1988). Organization of the *Ly5* gene.. Mol. Cell. Biol. **8:** 4889–4895.

Saiki R.K., Gelfand D.H., Stoffel S., Scharf S.J., Higuchi R., Horn G.T., Mullis K.B., and Erlich H.A. (1988). Primer directed enzymatic amplification of DNA with a thermostable DNA polymerase. Science **239:** 487–491.

Sarmiento M., Liken M.R., Trowbridge I.S., Coffman R.L., and Fitch F.W. (1982). High molecular weight lymphocyte surface proteins are structurally related and are expressed on different cell populations at different times during lymphocyte maturation and differentiation. J. Immunol. **128:** 1676–1684.

Scheid M.P., and Triglia D. (1979). Further description of the *Ly5* system. Immunogenetics **9:** 423–433.

Seldin M.F., D'Hoostelaere L.A., Steinberg A.D., Saga Y., and Morse H.C. (1987). Allelic variants of *Ly5* in inbred and natural populations of mice. Immunogenetics **26:** 74–78.

Shackelford D.A., and Trowbridge I.S. (1986). Identification of lymphocyte integral membrane proteins as substrates for protein kianse C. J. Biol. Chem. **261:** 8334–8341.

Shen F.-W. (1981). Monoclonal antibodies to mouse lymphocyte differentiation alloantigens. *In*: Monoclonal Antibodies and T Cell Hybridomas: Perspectives and Technical Advances, Hammerling, G.J., Hammerling U., and Kearny J.F., Eds. (Amsterdam: Elsevier/North-Holland), pp. 25–31.

Sparrow R.L., and McKenzie I.F.C. (1983). A function for human T200 in natural killer cytolysis. Transplantation **36:** 166–171.

Thomas M.L., Barclay A.N., Gagnon J., and Williams A.F. (1985). Evidence from cDNA clones that the rat leukocyte common antigen (T200) spans the lipid bilayer and contains a cytoplasmic domain of 80,000 $M_r$. Cell **41:** 83–93.

Thomas M.L., Reynolds P.J., Chain A., Ben-Neriah Y., and Trowbridge I.S. (1987). B cell variant of mouse T200 (*Ly5*): Evidence for alternative mRNA splicing. Proc. Natl. Acad. Sci. USA **84:** 5360–5363.

Thomas M.L., and Lefrancois L. (1988). Differential expression of the leucocyte-common antigen family. Immunol. Today **9:** 320–326.

Tonks N.K., Charbonneau H., Diltz C.D., Fischer E.H., and Walsh K.A. (1988). Demonstration that the leukocyte common antigen CD45 is a protein tyrosine phosphatase. Biochemistry **27:** 8695–8701.

Tung J.-S., Scheid M.P., Pierotti M.A., Hammerling U., and Boyse E.A. (1981). Structural features and selective expression of three *Ly5*$^+$ cell-surface molecules. Immunogenetics **14:** 101–106.

Tung J.-S., Saga Y., and Boyse E.A. (1988). Structural features of Ly-5 glycoproteins of the mouse and counterparts in other mammals. Immunogenetics **28:** 271–277.

Yakura H., Kawabata I., Shen F.-W., and Katagiri M. (1986). Selective inhibition of lipopolysaccharide-induced polyclonal IgG response by monoclonal *Ly5* antibody. J. Immunol. **136:** 2729–2733.

